# Different Impacts on the Heart After COVID-19 Infection and Vaccination: Insights From Cardiovascular Magnetic Resonance

**DOI:** 10.3389/fcvm.2022.916922

**Published:** 2022-07-14

**Authors:** Jan Gröschel, Yashraj Bhoyroo, Edyta Blaszczyk, Ralf Felix Trauzeddel, Darian Viezzer, Hadil Saad, Maximilian Fenski, Jeanette Schulz-Menger

**Affiliations:** ^1^Charité – Universitätsmedizin Berlin, Freie Universität Berlin, Humboldt-Universität zu Berlin, Working Group on Cardiovascular Magnetic Resonance, Experimental and Clinical Research Center, Berlin, Germany; ^2^Department of Cardiology and Nephrology, HELIOS Hospital Berlin-Buch, Berlin, Germany; ^3^German Centre for Cardiovascular Research (DZHK), Partner Site Berlin, Berlin, Germany; ^4^Department of Anaesthesiology and Intensive Care Medicine, Charité – Universitätsmedizin Berlin, Freie Universität Berlin und Humboldt-Universität zu Berlin, Berlin, Germany

**Keywords:** cardiovascular magnetic resonance, mapping, late gadolinium enhancement, COVID-19, vaccination, fibrosis

## Abstract

**Introduction:**

Myocarditis-like findings after COVID-19 (coronavirus disease 2019) infection and vaccination were reported by applying cardiovascular magnetic resonance (CMR). These results are very heterogenous and dependent on several factors such as hospital admission or outpatient treatment, timing of CMR, and symptomatic load. This retrospective study aimed to identify differences in myocardial damage in patients with persistent symptoms both after COVID-19 infection and vaccine by applying CMR.

**Materials and Methods:**

This study entails a retrospective analysis of consecutive patients referred for CMR between August 2020 and November 2021 with persistent symptoms after COVID-19 infection or vaccination. Patients were compared to healthy controls (HC). All patients underwent a CMR examination in a 1.5-T scanner with a scan protocol including: cine imaging for biventricular function and strain assessment using feature tracking, T2 mapping for the quantification of edema, and T1 mapping for diffuse fibrosis and late gadolinium enhancement (LGE) for the detection and quantification of focal fibrosis. Patients were divided into a subacute COVID-19 (sCov) group with symptoms lasting < 12 weeks, post-COVID-19 (pCov) group with symptoms > 12 weeks, and patients after COVID-19 vaccination (CovVac).

**Results:**

A total of 162 patients were recruited of whom 141 were included for analysis. The median age in years (interquartile range (IQR)) of the entire cohort was 45 (37–56) which included 83 women and 58 men. Subgroups were as follows (total patients per subgroup, median age in years (IQR), main gender): 34 sCov, 43 (37–52), 19 women; 63 pCov, 52 (39–58), 43 women; 44 CovVac, 43 (32–56), 23 men; 44 HC (41 (28–52), 24 women). The biventricular function was preserved and revealed no differences between the groups. No active inflammation was detected by T2 mapping. Global T1 values were higher in pCov in comparison with HC (median (IQR) in ms: pCov 1002ms (981–1023) vs. HC 987ms (963–1009; *p* = 0.005) with other parings revealing no differences. In 49/141 (34.6%) of patients, focal fibrosis was detectable with the majority having a non-ischemic pattern (43/141; 30.4%; patients) with the subgroups after infection having more often a subepicardial pattern compared with CovVac (total (% of group): sCov: 7/34(21%); pCov 13/63(21%); CovVac 2/44(5%); *p* = 0.04).

**Conclusion:**

Patients after COVID-19 infection showed more focal fibrosis in comparison with patients after COVID-19 vaccination without alterations in the biventricular function.

## Introduction

**FIGURE 1:**
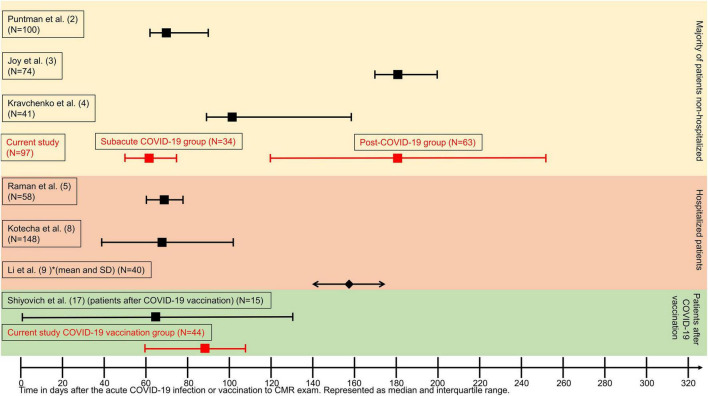
Graphical overview representing median time between COVID-19 infection or vaccination and cardiovascular magnetic resonance (CMR) examination. Time is represented as days on the X-axis. Data are given as median (squares) and interquartile range (whiskers indicate 25th and 75th percentile, respectively) except for Li et al. (9), which are represented as mean (diamond) and standard deviation (arrows pointing outward). Red colors highlight the time ranges of this study.

COVID-19 (coronavirus disease 2019) can virtually impact any organ ranging from the respiratory tract to the kidneys, the central nervous system, and the cardiovascular system (1). Similar to the broad range of organ involvement, the specific organ-related pathophysiologic changes can also show a wide array of patterns. Acute and mid-term myocardial tissue changes after COVID-19 infection have been described with varying degrees and frequencies, depending on various co-factors, such as hospitalization (2) or ambulatory recovery (3), timing between event and cardiovascular magnetic resonance (CMR) (3–5), and each individual’s risk factor profile (6–8). Taking the time between the acute event and CMR into consideration, patients can have reduced left (LV) and right ventricular (RV) function if examined within 2–3 months (2) or no biventricular impairment if CMR is performed 5 months after the initial event (9). Figure 1 visually integrates and compares this study with other published work regarding the time interval between CMR and acute infection or vaccination. Another factor to consider is the presence of symptoms, as evidence is expanding that they can have a high longevity even after the acute phase of the infection has subsided (10). Based on these findings, the terms subacute COVID-19 or long-COVID-19 for symptom persistency after 4 weeks of the infection and post-COVID-19 with ongoing symptoms for more than 12 weeks have been introduced by Nalbandian et al. in 2021 (10). From a cardiologic perspective, this is relevant as symptoms warranting further dedicated cardiologic work-up, such as fatigue, palpitations, and chest pain, are fairly common in these patients (11). One study recently reported findings in a patient cohort with ongoing symptoms, such as exertional dyspnea, fatigue, and palpitations, for more than 30 days after initial COVID-19 diagnosis (4). The studied population underwent a CMR examination at a median of 103 days revealing no signs of active myocardial inflammation in comparison with a healthy cohort. This raises the question how responsible structural myocardial impairment could actually be in terms of the symptom load or whether the etiology is more centered around a chronic fatigue syndrome with a complex neurological background. The first reports describe diagnostic criteria fulfillment for chronic fatigue syndrome in about half of the patients with ongoing symptoms after COVID-19 infection (12). CMR has been characterized as the non-invasive modality of choice for the detection of acute myocarditis (13) and is listed as a mandatory test in patients with heart failure and suspected myocarditis by the European Society of Cardiology (14). Even beyond the acute stages dominated by myocardial inflammation and edema, CMR can further deduce whether there is a complete recovery or whether changes might be persistent as marked by chronic replacement fibrosis detected on late gadolinium enhancement imaging (LGE) (15). Parametric techniques, such as T1 mapping and extracellular volume (ECV), might further identify potential diffuse fibrotic processes (13). Therefore, CMR might be useful in the assessment of patients after COVID-19 infection at different phases (16).

Along with the development of messenger RNA-based vaccines targeting the COVID-19 virus, reports on post-vaccination myocarditis followed (17, 18). A recent study of 15 patients undergoing CMR for clinically diagnosed post-vaccination myocarditis revealed findings similar to viral myocarditis. The patient cohort had a good clinical outcome (19). This was supported by another recent study demonstrating that patients with COVID-19 vaccination-associated myocarditis had no adverse outcomes and good clinical recovery (20). In comparison with patients with COVID-19 myocarditis and other viral myocarditis cases, the patients after COVID-19 vaccination showed less extensive LGE.

The focus of the study was on continuously symptomatic patients after COVID-19 infection or vaccination who were referred in an ambulatory setting for CMR. The aim of the investigation was to detect alterations in myocardial function and tissue structure with an intergroup comparison of patients with subacute COVID-19, post-COVID-19, and after COVID-19 vaccination.

## Materials and Methods

### Study Patients

**FIGURE 2:**
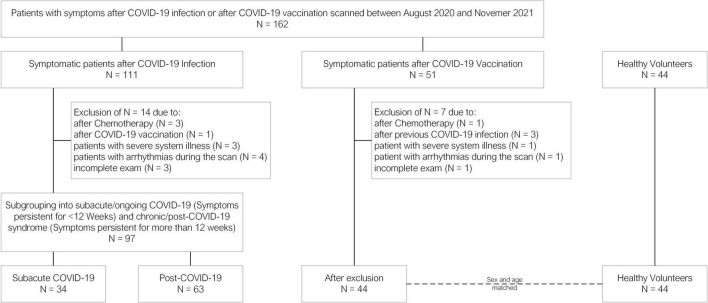
Flowchart detailing the patients excluded for each subgroup.

For this exploratory, retrospective study, all patients undergoing CMR examinations between August 2020 and November 2021 with persistent symptoms after either COVID-19 infection or vaccination were included. Patients were referred by primary care physicians or cardiologists. For the purposes of cohort characterization, the electronic health records were searched. Symptoms were systematically recorded before every scan by the attending physician on a standardized patient information sheet. After the inclusion, patients were subdivided into a cohort after COVID-19 infection with symptoms lasting between 4 and 12 weeks after infection (subacute COVID-19; sCov), with symptoms lasting > 12 weeks (post-COVID-19; pCov), and symptomatic patients after COVID-19 vaccination (CovVac). The time of the acute event was defined by the first positive polymerase chain reaction test or the time of the last dose of vaccination before symptom onset. Patients were excluded from the final analysis if severe systemic illnesses including systemic autoimmune disease, malignancies, cardiomyopathies, previous myocarditis, or previous chemotherapy were known. Similarly, patients who were vaccinated after COVID-19 infection were excluded and vice versa. Finally, if arrhythmias during the scan impaired image acquisition or the examination was incomplete, patients were excluded. A flowchart is shown in Figure 2. A healthy cohort (HC), recruited in previous studies before the outbreak of COVID-19, was age- and gender-matched to the CovVac patients and gender-matched to the sCov and pCov groups (15, 21). As only in a minority of HC contrast medium was applied, post-contrast image analysis was not carried out in the HC.

#### Ethics Statement

This study complied with the Declaration of Helsinki and was approved by the institutional ethics committee. Parts of the study were carried out under the PA-COVID study approval (clinicaltrials.gov: NCT04747366). The remaining patients were examined with the requirement for written informed consent being waived due to the retrospective study design (EA1/042/22).

### Cardiovascular Magnetic Resonance Imaging Protocol

All patients underwent a CMR examination on a 1.5-T scanner (AvantoFit^®^, Siemens, Erlangen, Germany) with ECG gating and a 32-channel surface phased-array coil. For the biventricular function assessment, balanced steady-state free precession cine images were acquired in four long-axis views including a four-, two-, three-chamber view as well as a RV view and one short-axis (SAX) stack, covering the entire ventricle without a gap. Parametric T2 and T1 mapping was acquired in multiple SAX slices covering the entire ventricle. T2-mapping acquisition was based on a motion-corrected balanced steady-state free precession sequence. In addition, T2-weighted imaging with a STIR (short T1 inversion recovery) sequence was carried out. Native T1 mapping was based on a motion-corrected modified Look-Locker inversion recovery technique using a 5–3–3 scheme. Synthetic ECV was calculated from T1-mapping pre- and post-contrast media application based on a prototype sequence in basal and midventricular slices. LGE imaging was acquired by a phase-sensitive inversion recovery sequence, 10–15 min after the application of 0.2mmol/kg of contrast media (gadoteridol, Prohance^®^, Bracco Imaging, Konstanz, Germany). LGE images were acquired in four-, two-, and three-chamber views as well as one SAX stack. Supplementary Material E1 shows a graphical representation of the full coverage approach for mapping and LGE acquisitions. Details about the sequence parameters are given in Supplementary Material E2.

### Cardiovascular Magnetic Resonance Image Analysis

Two readers [one with 6 years of experience in CMR (YB) and one with 2 years of experience (JG)] performed image analysis by using CVI42^®^ (version 5.13.0, Circle Cardiovascular Imaging, Calgary, Canada). The biventricular function assessment was executed on cine SAX images according to current recommendations (22). For the LV function assessment, papillary muscles were attributed to the total myocardial mass in diastole and systole. Left atrial function was assessed in cine four- and two-chamber views with a biplanar approach. Myocardial deformation assessment by feature tracking (FT) was carried out as published recently (23). STIR images were visually analyzed for myocardial edema. Quantitative mapping analysis was carried out with endo- and epicardial border delineation in each slice to obtain both global and segmental values, according to the 17-segment American Heart Association model, omitting the apical cap. Slice locations were allocated in the respective segment and level by delineating the extent of the LV. Slices with visible LV-outflow tract were excluded. Similarly, apical slices with no blood pool or thin myocardial walls were excluded. Institutional reference values for parametric mapping are as follows: native T1 (in ms) > 1018 (range 1018–1051), T2 (in ms) 52 (range 52–54), and ECV (in %) > 24 (24–30). Based on these cutoffs, the mean values and segmental values were categorized as normal or abnormal to assess differences in rates of abnormal mean and affected segments. A qualitative survey ensured to exclude segments with artifacts as well as focal fibrosis detected by LGE in order to properly assess diffuse fibrosis without confounding by focal replacement fibrosis. Focal scars were assessed visually by LGE analysis by both readers independently regarding the presence and location of scars. In case of uncertainties, a consensus read was performed. For LGE quantification, a semi-automated signal threshold versus reference mean method was chosen as previously described (24). Given the high frequency of non-ischemic scar burden, a 5-standard-deviation approach was applied (9, 25).

### Statistical Analysis

Continuous variables are expressed as mean and interquartile range. Categorical variables are given as absolute frequencies and percentages. Normal distribution was assessed by the Shapiro–Wilk test. Continuous variables were compared using either the Kruskal–Wallis method or one-way ANOVA. The correlation was based on the Spearman’s correlation coefficient given non-normal distribution. Categorical variables were compared using chi-square or Fisher’s exact test. A mixed model was used to assess differences regarding the rates of affected segments between the groups. In case of a significant global test, pairwise comparisons were performed. As all analyses were regarded exploratory, a significance level of 5% was regarded as a strong trend and was followed up by pairwise comparison with appropriate tests dispending adjustments for multiple comparisons. Intra- and interobserver agreement was assessed by Bland–Altman analysis based on 10 randomly chosen cases by JG and a third reader (MF; 4 years of experience in CMR), respectively. A *p*-value < 0.05 was considered statistically significant. Statistical calculations were performed using SPSS Statistics (version 27.0.0, IBM, Armonk, NY, United States) and SAS (version 9.4, SAS Institute Inc., Cary, NC, United States).

## Results

### Patient Characteristics

A total of 162 patients were recruited of whom 141 could be included for analysis (median age [interquartile range (IQR)], 45 (37–56); 83 women; 34/141 sCov, 43 (37–52); 19 women; 63/141 pCov, 52 (39–58); 43 women; 44/141 CovVac, 43 (32–56), 23 men; and 44 HC, 41 (28–52); 24 women; Table 1). Based on the group allocation on symptom duration, the time between infection and CMR was longer in the pCov group in comparison with the sCov group (median and interquartile range: pCov 180 (124–253) days vs. sCov 61 (50–76) days (*p* = < 0.001). This was similarily observable for the pCov and CovVac groups (pCov vs. CovVac 88 (60–107) days (*p* = < 0.001)). There was a difference regarding age between sCov and HC (*p* = 0.03) as well as pCov and HC (*p* = 0.01). In comparison with the HC, the three patient cohorts showed higher weight (sCov vs HC *p* = 0.047; pCov vs HC *p* = 0.03; CovVac vs HC *p* = 0.003) and body mass index (sCov vs HC *p* = 0.02; pCov vs HC *p* = < 0.001; CovVac vs HC *p* = 0.001). Comorbidities were equally distributed among the patient groups showing no differences, with arterial hypertension being the most common. In comparison with the CovVac group, patients after COVID-19 infection presented more often with ongoing fatigue (sCov 19/34 patients (56%) vs. CovVac 14/44 patients (32%; *p* = 0.03); pCov 38/63 patients (60%) vs. CovVac (*p* = 0.003); sCov vs. pCov (*p* = 0.07)) and palpitations (sCov 13/34 patients (38%) vs. CovVac 8/44 patients (18%; *p* = 0.047); pCov 26/63 patients (41%) vs. CovVac (*p* = 0.02); sCov vs. pCov (*p* = 0.77)). Systolic and diastolic blood pressure measurements during the scan revealed no differences. Higher heart rates were detected for pCov and CovVac patients in comparison with HC (pCov 74 (67–80) vs. HC 69 (61–75); *p* = 0.001); CovVac 74 (66–83) vs. HC (*p* = 0.02)). Dyspnea was observed more often in the pCov group compared with the CovVac patients (pCov 43/63 patients (68%) vs. CovVac 16/44 patients (36%; *p* = 0.001); sCov 17/34 patients (50%) vs. CovVac (*p* = 0.23); pCov vs. sCov (*p* = 0.77)). In total, 25 laboratory results for NT-pro-BNP and high-sensitive (hs) troponin-T were available: 8 in the sCov group (mean NT-pro-BNP in ng/L (IQR) 91 (32–103), mean hs troponin-T in ng/L (IQR) 6 (3–10)); 11 in the pCov group (NT-pro-BNP 66 (37–90), hs troponin-T 6 (3–6)); and 6 in the CovVac group (NT-pro-BNP 15 (4–22), hs troponin-T 4 (3–5)). There were no differences between hs troponin-T values but significant differences between sCov and CovVac (*p* = 0.01) as well as pCov and CovVac (*p* = < 0.001) regarding NT-pro-BNP levels. Patient characteristics are given in Table 1. In total, 14 patients were excluded from the COVID-19 infection group and seven from the CovVac group (see flowchart in Figure 2). No patient required hospitalization for ongoing symptoms. In the infection groups, 3/97 (3%) required hospitalization and one patient had to be admitted to the intensive care unit during the acute phase. None of the patients from the CovVac group required hospitalization. In the CovVac group, 40/44 patients (91%) received a messenger RNA-based vaccine and 4/44 (9%) received a vector-based vaccine. Of the 40 patients receiving an mRNA vaccine, 37/44 (84%) received BNT162b2 mRNA vaccine (Pfizer-BioNTech) and 3/44 (7%) received mRNA-1273 vaccine (Moderna). The majority of patients (37/44; 84%) presented after the first vaccination dose and 7/44 patients (16%) after the second dose.

**TABLE 1:** Summary of patient characteristics.

Parameter	All patients after COVID-19 infection (*N* = 97)	Subacute COVID-19 (*N* = 34)	Post-COVID-19 (*N* = 63)	COVID-19 vaccination (*N* = 44)	Healthy controls (*N* = 44)	*p* value*	Pairings with significant differences
Gender (F/M)	62/35	19/15	43/20	21/23	24/20	0.18	n.a.
Age (years)	48 (38–56)	43 (37–52)	52 (39–58)	43 (32–56)	41 (28–52)	0.02	**sCov vs. HC; pCov vs. HC**
Height (cm)	171 (164–180)	173 (166–181)	170 (163–180)	175 (167–182)	173 (168–180)	0.34	n.a
Weight (kg)	77 (65–86)	75 (67–85)	77 (64–86)	82 (65–97)	70 (63–78)	**0.02**	**sCov vs. HC; pCov vs. HC; CovVac vs. HC**
Body mass index (kg/m^2^)	25.3 (22.9–28.7)	24.9 (22.8–27.5)	25.5 (23.5–29.3)	25.8 (22.7–30.2)	22.9 (21–25.2)	**0.001**	**sCov vs. HC; pCov vs. HC; CovVac vs. HC**
Event to CMR (days)	141 (80–231)	61 (50–76)	180 (124–253)	88 (60–107)	n.a.	**<0.001**	**sCov vs. pCov; pCov vs. CovVac**
Heart rate (beats per minute)	74 (66–80)	72 (64–81)	74 (67–80)	74 (66–83)	69 (61–75)	**0.035**	**pCov vs. HC; CovVac vs. HC**
Systolic blood pressure (mmHg)	126 (115–132)	125 (118–130)	126 (115–134)	129 (117–137)	119 (113–135)	0.38	n.a.
Diastolic blood pressure (mmHg)	75 (70–84)	81 (72–89)	72 (70–83)	73 (68–80)	72 (67–77)	0.13	n.a.
Symptoms							
Fatigue	57 (58%)	19 (56%)	38 (60%)	14 (32%)	n.a.	**0.01**	**sCov vs. CovVac; pCov vs. CovVac**
Dyspnea	59 (60%)	17 (50%)	43 (68%)	16 (36%)	n.a.	**0.004**	**pCov vs. HC**
Chest pain	33 (34%)	13 (38%)	24 (38%)	21 (48%)	n.a.	0.43	n.a
Palpitations	36 (37%)	13 (38%)	26 (41%)	8 (18%)	n.a.	**0.04**	**sCov vs. CovVac; pCov vs. CovVac**
Comorbidities							
Arterial hypertension	27 (28%)	7 (21%)	20 (32%)	15 (34%)	n.a.	0.34	n.a.
Diabetes mellitus	4 (4%)	1 (3%)	3 (5%)	3 (7%)	n.a.	0.79	n.a.
Hyperlipidemia	8 (8%)	3 (9%)	5 (8%)	4 (9%)	n.a.	0.99	n.a.
Congestive heart failure	1 (1%)	1 (3%)	0 (0%)	2 (5%)	n.a.	0.17	n.a.
Coronary artery disease	3 (3%)	2 (6%)	1 (2%)	0 (0%)	n.a.	0.24	n.a.
Mild/moderate systemic disease	5 (5%)	1 (3%)	4 (6%)	1 (2%)	n.a.	0.66	n.a.
Chronic lung disease	4 (4%)	2 (6%)	2 (3%)	1 (2%)	n.a.	0.72	n.a.
Valvular heart disease	2 (2%)	1 (3%)	1 (2%)	3 (7%)	n.a.	0.44	n.a.
Chronic kidney disease	0 (0%)	0 (0%)	0 (0%)	1 (2%)	n.a.	0.55	n.a.

*Data are median and interquartile ranges for continuous and number with percentages in brackets for continuous variables. p < 0.05 is considered to indicate a statistically significant difference.*

*COVID-19, coronavirus disease 2019; sCov, subacute COVID-19; pCov, post-COVID-19; CovVac, COVID-19 vaccination; HC, healthy controls.*

*p-values given for tests between subacute COVID-19, post-COVID-19, COVID-19 vaccination, and healthy controls.

*Bold text represents statistically significant differences.*

*n.a., not applicable.*

### Cardiovascular Magnetic Resonance Results

The biventricular function was within normal ranges for the entire studied population with no differences in LV ejection fraction (EF; sCov 61.6% (56.8–65.6); pCov 62.6% (59.2–65.7); CovVac 61.7% (56.7–63.9); HC 62.3% (58–66.2; *p* = 0.46)) and RV-EF (sCov 53.8% (50.6–56.7); pCov 53.6% (48.3–57.6); CovVac 52.1% (47.3–55.7); HC 52.9% (50.1–58.9; *p* = 0.43)). Global radial and circumferential strains were lower in the patient cohorts in comparison with the HC (see Table 2), but after exclusion of patients with focal scars on LGE, no differences between the groups were detectable for global radial strain (sCov 25.9% (24.1–30.7); pCov 26.1% (23.7–29.3); CovVac 26.2% (22.3–28.7); HC 29.1% (26–30.3; *p* = 0.07)) and global circumferential strain (sCov –16.7% (−18.7 – (−16)); pCov −16.8%(−18.1 – (−15.8)); CovVac −16.8% (−17.7 – (−15)); HC −18% (−18.5 – (−16.7); *p* = 0.07)). Global longitudinal strain values did not show significant differences between the groups (sCov −18.6 (−20.4 – (−16.4)); pCov −18 (−19.3 – (−16.4)); CovVac −17.5 (−19.5 – (−15.4)); HC −18 (−19.1 – (−16.9); *p* = 0.52)). T2-weighted imaging revealed no myocardial edema. Pericardial effusions were detected in 50 patients (sCov 15/34 (44%); pCov 25/63 (40%); CovVac 10/44 (23%; *p* = 0.09)). None of them were hemodynamically relevant.

**TABLE 2:** Cardiac function parameters derived from cardiovascular magnetic resonance (CMR).

Parameter	All patients after COVID-19 infection (*N* = 97)	Subacute COVID-19 (*N* = 34)	Post-COVID-19 (*N* = 63)	COVID-19 vaccination (*N* = 44)	Healthy controls (*N* = 44)	*p* value*	Pairings with significant differences
LV-EDV (ml)	141.8 (121.6–168.2)	143.6 (124.9–172.8)	137.6 (118.8–167.2)	162.1 (126.6–193.2)	138.6 (119.7–162.8)	0.16	n.a.
LV-ESV (ml)	53.6 (43.4–66.8)	54 (45–75.1)	51.9 (42.3–62.5)	59 (47.8–74.1)	52.2 (43.7–64.8)	0.15	n.a.
LV-SV (ml)	87.7 (75.1–103.1)	88.2 (73.9–103.9)	86.6 (75.1–104.3)	97.5 (79.3–110.6)	84.6 (74.4–100.8)	0.64	n.a.
LV-EF (%)	62.3 (58.5–65.6)	61.6 (56.8–65.6)	62.6 (59.2–65.7)	61.7 (56.7–63.9)	62.3 (58–66.2)	0.46	n.a.
LVM (g)	80.8 (66.5–103.5)	81.1 (68.5–108)	80.8 (65.7–102.8)	97.7 (74.6–115.6)	82.3 (69.4–99.8)	0.09	n.a.
RV-EDV (ml)	151.5 (132.4–183.5)	154.6 (134.4–193.6)	151.3 (129.8–178.9)	174.4 (132–204.7)	160.4 (138.9–182.9)	0.58	n.a.
RV-SV (ml)	82.3 (71.4–95.5)	83.2 (72–103.4)	78.8 (71.3–95.1)	92.1 (72.2–102.4)	83.4 (73.4–99.2)	0.53	n.a.
RV-EF (%)	53.6 (49.6–57.1)	53.8 (50.6–56.7)	53.6 (48.3–57.6)	52.1 (47.3–55.7)	52.9 (50.1–58.9)	0.43	n.a.
LA (cm^2^)	20 (17.4–22.3)	20 (16.7–22.4)	20 (17.5–22.6)	20.7 (18.6–23.2)	20.9 (18.7–22)	0.71	n.a.
LA-EF (%)	65.1 (59–70.4)	68.6 (58.1–72.9)	63.6 (59.1–67.8)	63.9 (60.4–71.6)	61.7 (58.3–69.2)	0.06	n.a.
LA-EDV (ml)	60 (49.5–72.7)	58.8 (48.9–71.6)	60.1 (49–73.7)	64.8 (53.4–75.1)	61.9 (51.6–68.9)	0.53	n.a.
LA-SV (ml)	38.3 (31.2–47.9)	38.7 (31–50.4)	37.5 (30.9–47.8)	42.9 (33.5–50)	39.4 (31.6–43)	0.32	n.a.
GLS (%)	−18.3 (−19.8–(−16.4))	−18.6 (−20.4–(−16.4))	−18 (−19.3–(−16.4))	−17.5 (−19.5–(−15.4))	−18 (−19.1–(−16.9))	0.52	n.a.
GRS (%)	25.7 (23–28.7)	25.9 (22.5–29.4)	25.7 (23.1–28.5)	25.7 (22–28.8)	29.1 (26–30.3)	**0.004**	**sCov vs. HC; pCov vs. HC; CovVac vs. HC**
GCS (%)	−16.7 (−17.9–(−15.4))	−16.7 (−18–(−15.2))	−16.7 (−17.8–(−15.5))	−16.7 (−17.6–(−15))	−18 (−18.5–(−16.7))	**0.005**	**sCov vs. HC; pCov vs. HC; CovVac vs. HC**
GRS (%) without LGE + patients	26.1 (24–29.4) (*N* = 50)	25.9 (24.1–30.7) (*N* = 23)	26.1 (23.7–29.3) (*N* = 37)	26.2 (22.3–28.7) (*N* = 28)	29.1 (26–30.3) (*N* = 44)	0.07	n.a.
GCS (%) without LGE + patients	−16.8 (−18.1–(−16)) (*N* = 50)	−16.7 (−18.7–(−16) (*N* = 23)	−16.8 (−18.1–(−15.8)) (*N* = 37)	−16.8 (−17.7–(−15)) (*N* = 28)	−18 (−18.5–(−16.7)) (*N* = 44)	0.07	n.a.

*Data are median and interquartile ranges. p < 0.05 is considered to indicate a statistically significant difference.*

*COVID-19, coronavirus disease 2019; LV-EDV, left ventricular end-diastolic volume; LV-ESV, left ventricular end-systolic volume; LV-SV, left ventricular stroke volume; LV-EF, left ventricular ejection fraction; LVM, left ventricular mass; RV-EDV, right ventricular end-diastolic volume; RV-ESV, right ventricular end-systolic volume; RV-SV, right ventricular stroke volume; RV-EF, right ventricular ejection fraction; LA, left atrium; LA-EF, left atrial ejection fraction; LA-EDV, left atrial end-diastolic volume; LV-SV, left atrial stroke volume; GLS, global longitudinal strain; GRS, global radial strain; GCS, global circumferential strain; sCov, subacute COVID-19; pCov, post-COVID-19; CovVac, COVID-19 vaccination; HC, healthy controls.*

*p-values given for tests between subacute COVID-19, post-COVID-19, COVID-19 vaccination, and healthy controls.

*Bold text represents statistically significant differences.*

*n.a., not applicable.*

**FIGURE 3:**
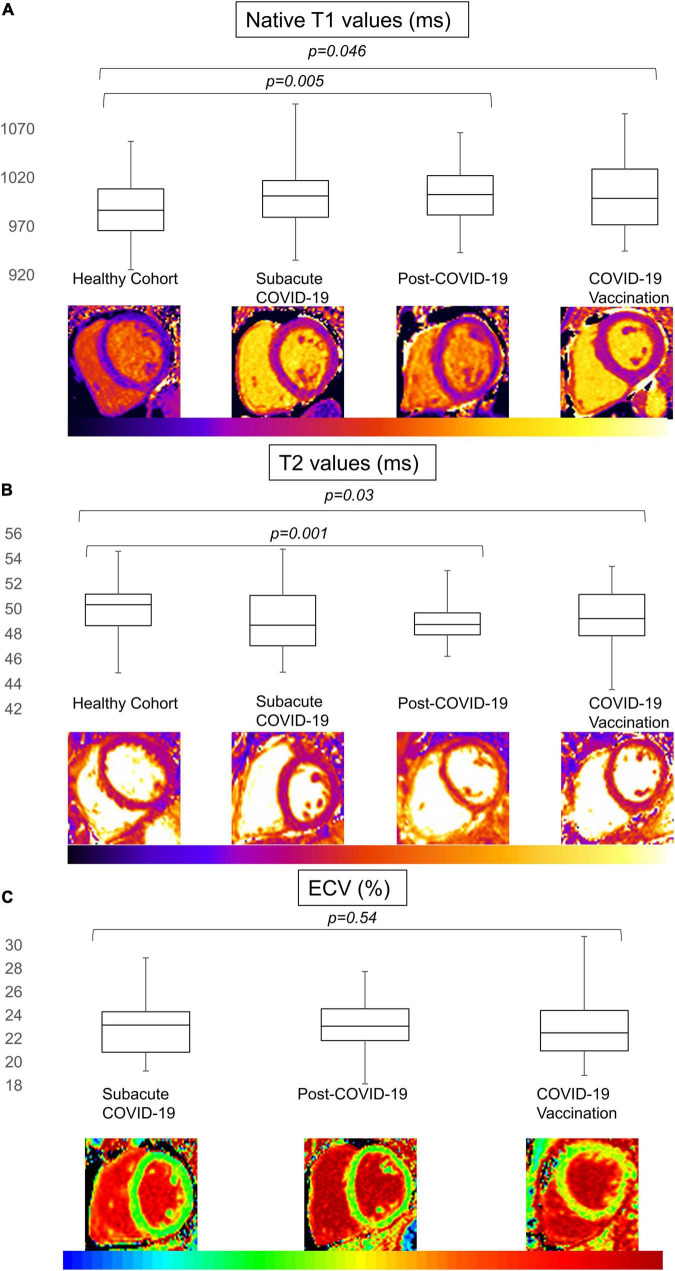
T1, T2, and extracellular volume (ECV) values for the patient cohort and healthy controls. Boxplot representation of the mapping values for T1 in ms **(A)**, T2 in ms **(B)**, and ECV in% **(C)** for patients after COVID-19 infection (subacute and post-COVID-19), after COVID-19 vaccination, and healthy controls (from left to right in each panel). Whiskers represent minimal and maximal values with boxes representing 25th percentile, median, and 75th percentile (from bottom to top). Significant values for general tests were followed by subgroup comparison. A *p*-value of < 0.05 was regarded as statistically significant.

Global native T1 mapping did not differ between HC, sCov, and CovVac, whereas pCov patients showed higher global values in comparison with HC (pCov 1002 ms (981–1023) vs. HC 987 ms (963–1009; *p* = 0.005); Table 3). Basal native T1 values were higher in the pCov and CovVac groups in comparison with HC (pCov 1008 ms (990–1022) vs HC 993 ms (972–1014; *p* = 0.005); CovVac 1006 ms (975–1032) vs HC (*p* = 0.02)). Admittedly, no patients presented with signs of active inflammation, but differences were found between pCov patients and the HC for global T2 times (pCov 48.8 ms (47.9–49.8) vs. HC 50.4 ms (48.5–51.2; *p* = 0.001)), basal (pCov 48.2 ms (47.1–49.3) vs. HC 50.1 ms (47.6–50.8; *p* = 0.01)), and midventricular T2 slices (pCov 48.7 ms (47.8–49.6) vs. HC 50.2 ms (48.3–51.2; *p* = 0.001)). ECV showed no differences between the patient groups. Figure 3 visually represents the mapping findings. Based on the reference values given in methods section, we did not find a statistical difference for the rates of T1 involvement between the groups (patients with T1 above cutoff/total patients in the group (%): sCov 10/34 (29%); pCov 18/63 (29%); CovVac 15/44 (34%); and HC 5/44 (11%); *p* = 0.07). No statistically significant differences were found between the patient groups regarding ECV (patients with ECV above cutoff/total patients in the group (%): sCov 10/34 (29%); pCov 18/63 (29%); and CovVac 13/44 (30%) *p* = 0.99). Regarding rates of affected segments for T1, we found statistically significant differences between the groups for all segments (*p* = 0.02), basal (*p* = 0.04), and midventricular segments (*p* = 0.03). In a pairwise comparison, the differences were between sCov and HC for midventricular segments (rate difference of affected segments 0.105; *p* = 0.04), between pCov and HC for basal segments (rate difference of affected segments 0.09; *p* = 0.045), and between CovVac and HC for all T1 segments (rate difference of affected segments 0.142; *p* = 0.002), basal (rate difference of affected segments 0.144; *p* = 0.004), and midventricular segments (rate difference of affected segments 0.136; *p* = 0.005). We separately compared 14 older HC controls (age 54 years (49–63) to 14 age-, gender-, weight-, and height-matched pCov patients (age 56 years (49–64). No statistically significant differences were found for T1 times (pCov median 1014ms (982–1037); older HC median 994ms (977–1010); *p* = 0.09) and T2 times (pCov median 48.8 ms (48.1–50.7); and older HC median 50.8ms (50.1–51.2); *p* = 0.1). Details about the number of slices analyzed and segments excluded for parametric assessment are given in Supplementary Material (E3). Visual Bland–Altman revealed good intra- and interobserver agreement for functional and parametric assessment (Supplementary Material E4).

**TABLE 3:** Parametric mapping quantification derived by CMR.

Parameter	Subacute COVID-19 (*N* = 34)	Post-COVID-19 (*N* = 63)	COVID-19 vaccination (*N* = 44)	Healthy controls (*N* = 44)	*p* value*	Pairings with significant differences
T1 global (ms)	1001 (977–1029)	1002 (981–1023)	999 (968–1030)	987 (963–1009)	**0.046**	**pCov vs. HC**
T1 basal (ms)	1003 (980–1030)	1008 (990–1022)	1006 (975–1032)	993 (972–1014)	**0.04**	**pCov vs. HC; CovVac vs. HC**
T1 mid (ms)	1001 (976–1025)	999 (982–1027)	995 (973–1029)	987 (966–1010)	0.10	n.a.
T1 apical (ms)	987 (957–1034)	996 (969–1027)	992 (951–1038)	985 (962–1009)	0.66	n.a.
T2 global (ms)	48.7 (47–51.2)	48.8 (47.9–49.8)	49.2 (47.8–50.3)	50.4 (48.5–51.2)	**0.03**	**pCov vs. HC**
T2 basal (ms)	48.5 (46.6–50.3)	48.2 (47.1–49.3)	49.1 (47.5–50.3)	50.1 (47.6–50.8)	**0.03**	**pCov vs. HC**
T2 mid (ms)	48.8 (47–51)	48.7 (47.8–49.6)	49 (47.6–51)	50.2 (48.3–51.2)	**0.04**	**pCov vs. HC**
T2 apical (ms)	49.7 (47.2–52.2)	50 (48.4–51.1)	50.3 (48.3–52.7)	51.1 (48.5–52.1)	0.33	n.a.
ECV global (%)	23.2 (20.8–24.4)	23.1 (21.8–24.7)	22.5 (20.9–24.5)	n.a.	0.54	n.a.
ECV basal (%)	22.6 (20.8–24.4)	23 (21.5–24.3)	22.6 (20.6–24.3)	n.a.	0.47	n.a.
ECV mid (%)	22.9 (20.6–24.4)	23.4 (21.9–24.8)	22.8 (20.9–24.8)	n.a.	0.39	n.a.

*Data are median and interquartile ranges. p < 0.05 is considered to indicate a statistically significant difference.*

*COVID-19, coronavirus disease 2019; ECV, extracellular volume; sCov, subacute COVID-19; pCov, post-COVID-19; CovVac, COVID-19 vaccination; HC, healthy controls.*

*p-values given for tests between subacute COVID-19, post-COVID-19, COVID-19 vaccination, and healthy controls.

*Bold text represents statistically significant differences.*

*n.a., not applicable.*

**FIGURE 4:**
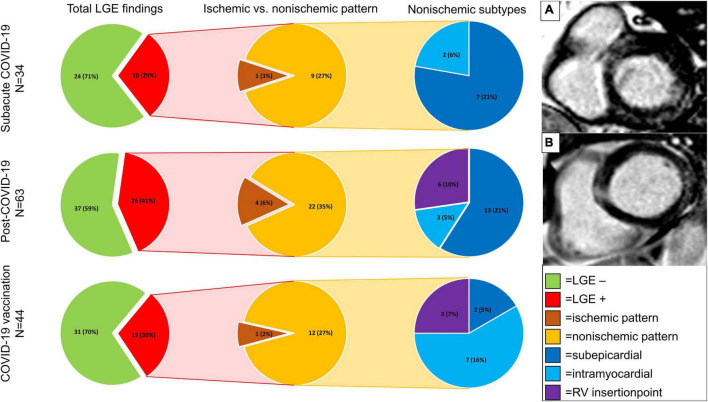
Focal fibrosis detected by late gadolinium enhancement imaging in the patient cohorts. Presented are total and percentages of findings (findings/cohort size) in pie charts. Different subtypes of late gadolinium enhancement (LGE) patterns are indicated by colors with a legend on the lower right side (lime green = no LGE; red = LGE positive; brown = ischemic pattern; orange = non-ischemic pattern; dark blue = subepicardial LGE; light blue = intramyocardial LGE; purple = RV insertion point). Significant differences were found between subepicardial LGE findings in the subacute COVID-19 group and the COVID-19 vaccination group (*p* = 0.04) and between the post-COVID-19 group and the COVID-19 vaccination group (*p* = < 0.001). No differences were found between the infection subgroups (*p* = 0.99) for subepicardial LGE. Other pairings revealed no differences. **(A)** Subepicardial scar in the basal inferolateral part. **(B)** Subepicardial scar in the basal lateral part.

Visual LGE analysis revealed focal scars in 49/141 patients (34.6%). There was no statistically significant difference between the groups regarding the rate of patients with LGE findings (sCov 10/34 (29%); pCov 26/63 (41%); CovVac 13/44 (30%; *p* = 0.34)). A non-ischemic pattern dominated in the entire study with 43/49 (88%) being either subepicardial, intramyocardial, or RV insertion point fibrosis (non-ischemic scars/total scars: sCov 9/10 (90%); pCov 22/26 (85%); CovVac 12/13 (92%). For sCov (7/10 (70%)) and pCov (13/26 (50%), a subepicardial pattern was most commonly encountered, whereas CovVac patients most often displayed an intramyocardial pattern (7/13 (54%)). In comparison with the CovVac group, patients after COVID-19 infection had more focal subepicardial findings (subepicardial fibrosis/patients per group: sCov 7/34 (21%) vs. CovVac 2/44 (5%; *p* = 0.04); pCov 13/63 (21%) vs. CovVac (*p* = < 0.001)); however, no differences were found between the subgroups after an infection (*p* = 0.99; Figure 4; details in Supplementary Material E5). In the sCov group, 6/7 (86%) of subepicardial scars were located in the basal segments with one in the anterolateral wall (1/7; 14%), four in the inferolateral wall (4/7; 57%), and one in the lateral wall (1/7; 14%). One subepicardial scar was found in the medial-lateral wall (1/7; 14%). The intramyocardial scars were in the middle ventricular section with one being in the septal and one in the lateral wall. For the pCov groups, all LGE findings were located in the basal part. Of the 13 subepicardial scars, six were found in the lateral segments (6/13; 46%), five in the inferolateral segments (5/13; 39%), and two in the inferior segments (2/13; 15%). Similarly, the intramyocardial scars were in a majority of cases in the lateral wall (2/3; 66%) with one in the inferolateral wall (1/3; 33%). The CovVac group had in total more intramyocardial scars with three in the inferior basal segment (3/7; 43%), three in the inferolateral basal segment (3/7; 43%), and one in the lateral basal segment (1/7; 14%). The two subepicardial findings were equally distributed in the basal part with one each in the lateral (1/2; 50%) and inferolateral segments (1/2; 50%). Statistically, there were no differences regarding the lateral (*p* = 0.34), inferolateral (*p* = 0.81), and inferior (*p* = 0.16) segments regarding the expected frequency of distribution between sCov, pCov, and CovVac groups. Of the six patients with ischemic scars, only one had a previous medical history of coronary artery disease. The majority of ischemic LGE lesions were found in the pCov group (4/6; 67%). In the sCov group, one patient (1/34; 3%) had a lateral subendocardial scar covering the basal to early apical segments. Of the four patients with ischemic scar burden in the pCov group (4/63; 6%), two had an anterior basal location, one had an inferior lateral pattern in the basal part, and the remaining patient had a small but visible scar in the apical region. One patient from the CovVac group had a lateral subendocardial scar in the basal segments (1/44; 2%). LGE quantification showed no difference between the groups, neither for total enhanced mass (*p* = 0.95) nor for enhanced percentage (*p* = 0.52; Table 4).

**TABLE 4:** Quantitative late gadolinium enhancement (LGE) findings.

Parameter	Total (*N* = 48)	All patients after COVID-19 infection (*N* = 35)	Subacute COVID-19 (*N* = 10)	Post-COVID-19 (*N* = 25)	COVID-19 vaccination (*N* = 13)	*p* value*	Pairings with significant differences
Total enhanced volume (ml)	1.4 (0.5–2.4)	1.4 (0.5–2.1)	0.9 (0.5–2.7)	1.7 (0.6–2)	1 (0.3–2.6)	0.94	n.a.
Total enhanced mass (g)	1.5 (0.5–2.5)	1.5 (0.6–2.2)	1 (0.5–2.8)	1.6 (0.6–2.1)	1.1 (0.3–2.7)	0.95	n.a.
Enhanced volume (%)	2 (0.9–4.1)	2.1 (1–4.1)	1.7 (0.9–5.9)	2.3 (1.3–4.1)	1.2 (0.4–4.5)	0.52	n.a.

*Data are given as median and interquartile range. p < 0.05 is considered to indicate a statistically significant difference.*

*COVID-19, coronavirus disease 2019; sCov, subacute COVID-19; pCov, post-COVID-19; CovVac, COVID-19 vaccination; HC, healthy controls.*

*p-values given for tests between subacute COVID-19, post-COVID-19, and COVID-19 vaccination.

*n.a., not applicable.*

No correlation between overall symptom load, defined as the sum of the symptoms (fatigue, dyspnea, chest pain, and palpitations), and markers of myocardial involvement, especially the presence of LGE (*r* (Spearman’s correlation coefficient) = 0.07), mean native T1 (*r* = 0.03), mean T2 (*r* = −0.17), and mean ECV (*r* = 0.13), was found. Similarly, no statistical differences were found between patients with no symptoms and patients with at least one symptom considering the entire patient cohort (mean native T1 *p* = 0.56; mean T2 *p* = 0.11; mean ECV *p* = 0.27).

## Discussion

The ongoing COVID-19 pandemic remains to be a burden for healthcare systems around the globe with symptoms persisting for more than half a year after an acute infection in some patients (11). In this retrospective analysis, we identified a higher focal fibrotic burden in patients with persistent symptoms after COVID-19 infection in comparison with patients after COVID-19 vaccination.

CMR analysis revealed normal biventricular function and no active myocardial inflammation. Global T2 times were lower in the pCov group compared with the HC. Regarding this finding, we can only speculate about its implication. Potential discrepancies in oxygen delivery to the myocardial tissue or a complex interaction between fibrosis and myocardial inflammation might be involved (26). Another explanation for the lower T2 times together with the higher native T1 times in pCov in comparison with HC could be the higher age in the pCov cohort. Previous studies on T1 values have reported an increase of around 12–15 ms per decade (27). In our subgroup comparison between the pCov and the older HC, we found no differences for T1 and T2 underlining these results. However, the subgroup only entails 14 cases of both groups limiting the generalizability of this non-significant finding. In addition, comparing the absolute values of T1 in the older subgroup (994 ms (range 977–1010) to the entire HC (987 ms (963–1009), the absolute differences are marginal. Next to age, other potential confounders could include the difference in weight and BMI between the groups as recent studies found significant associations between T1 times and weight (28). The overall small differences for T1 and T2 times are well within the limits of the intra- and interobserver limits of agreement (Supplementary Material E4). Therefore, these findings require further investigation in follow-up studies as well as multicenter studies to understand their full clinical impact. One other potential explanation might be that segments without chronic replacement fibrosis are undergoing long-lasting more subtle and diffuse changes that evolve over months. Several studies reported dynamics of T1 relaxation times over a time course of 6 months after an acute viral myocarditis (15, 29). It was shown that for viral myocarditis, T2 times might be elevated even up to 5 weeks after the acute event, but return to normal within 6 months, with T1 times behaving similarly with the exception that they might be elevated beyond the 6-month time frame (15). It is not clear yet whether the pathophysiologic and myocardial injury pattern after a COVID-19 infection differs from a classic viral myocarditis or whether the course is comparable. The current evidence is conflicting with one study reporting reduced T1 and elevated T2 times at follow-up examinations 68 days after the baseline scan (29). This contrasts with others, who reported no signs of active myocardial inflammation in patients with persistent symptoms (4). The latter findings are in line with ours as we also did not find evidence for an acute inflammatory process at the time of the CMR examination. The large Hamburg City Health Study COVID program reported findings in patients 9 months after the first positive test, comparing this group to healthy matched controls (30). They did not find any differences between patients and the healthy controls for T1 and T2 times. LGE findings were more predominant in the group after an infection but did not reach statistical significance (30). Similar to the conflicting evidence regarding T1 and T2, ECV findings also differ substantially. One group described persistently elevated ECV values (9), whereas Filipetti et al. showed that during follow-up, ECV as well as T1 times significantly decreased (31). Both studies analyzed patients after hospital admissions. We observed no difference in the sCov and pCov groups. Depending on the severity of the initial symptoms and the requirement for hospitalization, there might be either an improper immune response with persistent inflammation (2, 29) or a more subtle and diffuse process (9) that drives the changes after COVID-19. We did not, however, find a correlation between symptom load and myocardial tissue changes visualized by CMR for any patient group. This finding is supported by other studies which also did not find any correlation between reported symptoms and tissue changes (32, 33).

For the basal part, we found higher native T1 values for pCov and CovVac in comparison with the HC. These findings could potentially indicate a diffuse focal interstitial process. This is underlined by finding a higher rate of segmental involvement in all groups in comparison with healthy volunteers. Interestingly, in the CovVac cohort not only basal and midventricular slices were more often focally affected, but also the overall segmental affection rate was higher. In studies including patients after COVID-19 vaccination, findings were similar to a viral myocarditis but less pronounced (19, 20). One group reported a normal LV-EF, elevated T1 times in 46%, and LGE findings in 87% (19). The majority of LGE findings were found in the basal inferolateral region (19). The population in this study was clinically diagnosed and scanned at a median of 65 days (range 3–130) after the second dose. Fronza et al. presented findings for patients after COVID-19 vaccination myocarditis and COVID-19 infection with a mixed patient profile of hospitalized and non-hospitalized patients (20). Patients after the vaccination had higher LV-EF and lower native T1 values. In a short-term follow-up, LV-EF was further improving and no clinical adverse events were observed (20). In contrast to the above-mentioned studies, our population was scanned at a median of 88 days (IQR 60–107) after receiving a vaccination, likely reflecting a different stage. This is also shown by normal T2 times and the prevalence of LGE findings in our CovVac cohort with non-ischemic scars in 12/44 patients (27%). In comparison with the groups after COVID-19 infection, CovVac presented with less focal subepicardial scars. The frequency of subepicardial involvement in our study is higher than that of Kravchenko et al. (5%; all patients non-hospitalized) (4) but similar to Puntmann et al. (20%, 67% patients recovered at home) (2) and Kotecha et al. (22%, hospitalized patients) (8). The main segments involved were the inferior and inferolateral ones. This is in accordance with Wang et al. who, despite a more scattered pattern, found in 10 out of 12 patients subepicardial or intramyocardial LGE in these segments (34). It should be noted that these patterns are commonly described in cases with viral myocarditis (15, 35). As mentioned in the editorial by Lim and Bluemke (36), it has yet to be shown how the presence of LGE findings in symptomatic patients after acute COVID-19 infection might influence prognosis or relate to symptom load. Similarly, this holds true for symptomatic patients after COVID-19 vaccination. Strain analysis might potentially help to better understand myocardial dynamics after COVID-19 infection. One study with follow-up CMR performed at 3 months also detected reduced global circumferential strain in patients with LGE findings (34). It has been shown that strain assessment by FT correlates significantly with the ECV burden in patients with non-ischemic cardiomyopathies (37), potentially being a non-contrast dependent tissue marker for myocardial fibrosis. Strain values can also aid in risk stratification with decreased strain values being associated with worse outcomes (38, 39). Similar results have been observed by the application of strain-encoded magnetic resonance (SENC) tagging acquisitions (40). In contrast to strain analysis by FT, SENC relies on the additional acquisition of images. However, recent advances have reduced the necessary time to a single heartbeat with the possibility of free-breathing acquisitions in a technique called fast-SENC (41). Bucius et al. showed that despite a significant difference between FT and fast-SENC for the assessment of global strain values, there is an excellent agreement between these techniques (41). It should be noted that in the same study, FT had the lowest segmental inter-study agreement. Therefore, only global strain values are reported as regional strain values vary depending on number of slices, contouring, and post-processing software as described recently (23). Studies presenting follow-up data are required to further cohesively understand the pathophysiologic changes in the myocardium after acute COVID-19 infection and its sequelae and should base the results on the same standardized image analysis conditions.

Although there are significant differences regarding the NT-pro-BNP levels between the groups after infection in the CovVac group, we want to underline that first, the sample size is small compared with the entire cohort and second, all values are below the laboratory cutoff values/thresholds (NT-pro-BNP < 500 ng/dl and hs troponin-T < 15 ng/dl).

## Limitations

Our study has some limitations. First, there was a selection bias as only patients with symptoms were referred for CMR, omitting asymptomatic patients after COVID-19 infection or vaccination. Second, given the retrospective nature of our study, laboratory tests were available for only a minority of patients. Hence, the analysis of the laboratory tests only covers a subgroup. Similarly, no information was available regarding the medication at the time of the scan. Third, no intraindividual follow-up data can be presented at this time point. Fourth, the age difference between the healthy cohort and the two patient groups after COVID-19 infection could have potentially influenced the mapping results, as shown by the subgroup comparison. Finally, ECV and LGE cannot be provided in the healthy cohort as the application of contrast media was limited due to concerns from the ethical board.

## Conclusion

In summary, we conclude that all patients had a normal biventricular function, but more diffuse fibrosis was detectable in symptomatic patients after COVID-19 infection with symptom persistence for more than 12 weeks. This mandates further research into pathophysiologic and histopathological changes connected with COVID-19. In comparison with symptomatic patients after COVID-19 vaccination, more focal subepicardial scars were detected in patients after an infection with the COVID-19 virus.

## Data Availability Statement

The datasets presented in this article are not readily available because of German data privacy laws. Requests to access the datasets should be directed to the corresponding author.

## Ethics Statement

The studies involving human participants were reviewed and approved by Charité’s Ethics Committee (EA1/042/22). Written informed consent for participation was not required for this study in accordance with the national legislation and the institutional requirements.

## Author Contributions

JG and JS-M were the guarantors of integrity of entire study. JG, MF, and JS-M were involved in the literature research and were involved in the statistical analysis. All authors were involved in the study concepts/study design or data acquisition or data analysis/interpretation, manuscript drafting or manuscript revision for important intellectual content, approval of the final version of submitted manuscript, and manuscript editing and agreed to ensure any questions related to the work are appropriately resolved.

## Conflict of Interest

The authors declare that the research was conducted in the absence of any commercial or financial relationships that could be construed as a potential conflict of interest.

## Publisher’s Note

All claims expressed in this article are solely those of the authors and do not necessarily represent those of their affiliated organizations, or those of the publisher, the editors and the reviewers. Any product that may be evaluated in this article, or claim that may be made by its manufacturer, is not guaranteed or endorsed by the publisher.

## Abbreviations

COVID-19: Coronavirus disease 2019
CMR: cardiovascular magnetic resonance
HC: healthy controls
LV: left ventricle
RV: right ventricle
LGE: late gadolinium enhancement
ECV: extracellular volume
sCov: subacute COVID-19
pCov: post-COVID-19
CovVac: COVID-19 vaccination
SAX: short axis
STIR: short T1 inversion recovery
FT: feature tracking
IQR: interquartile range
hs: high sensitive
EF: ejection fraction
SENC: strain-encoded magnetic resonance.
