# Supplementary material for: Different Impacts on the Heart After COVID-19 Infection and Vaccination: Insights From Cardiovascular Magnetic Resonance
Source: Front Cardiovasc Med. 2022 Jul 14;9:916922. doi: 10.3389/fcvm.2022.916922 (PMC9329612; doi:10.3389/fcvm.2022.916922)
Supplement: Supplementary file 1 [file Data_Sheet_1.docx]

Supplementary Material

Supplementary Data 1. Approach to complete ventricular coverage for parametric T1 and T2-Mapping and late gadolinium enhancement.


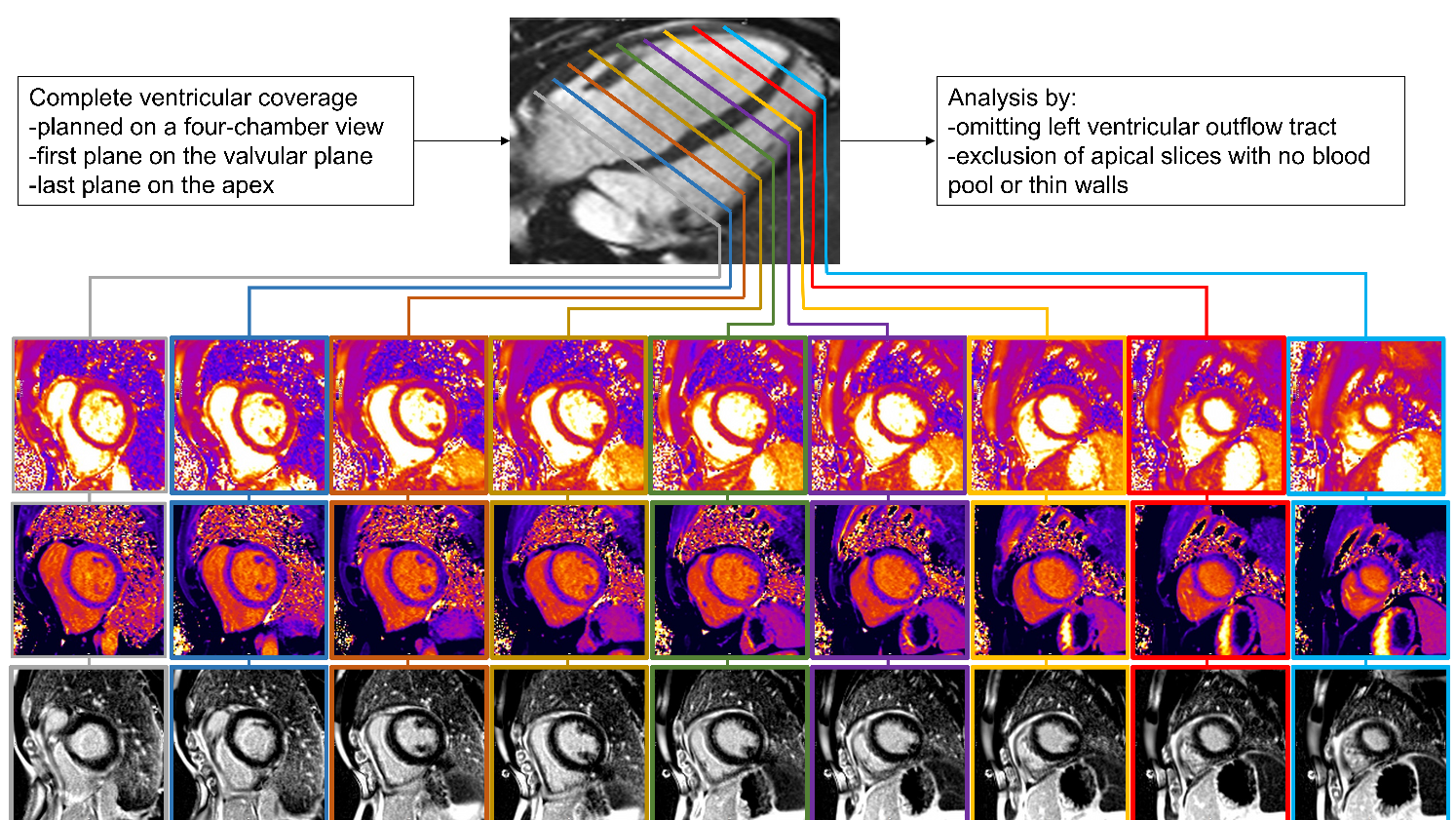


Slice localization was planned on a four-chamber view with the first slice covering the valvular plane. Left and right ventricle were covered till the apex. For mapping analysis slices with left ventricular outflow tract or artefacts as well as apical with no blood pool and/or thin walls were excluded. The left ventricular extent was determined by placement of a line between the mitral valve and the apex (not shown). Slices were then allocated to basal, midventricular and apical layers according to the American Heart Association model excluding the apical gap. Similarly in patients with focal fibrosis on late gadolinium enhancement images, scar tissue was quantified applying a 5 standard deviation approach, obtaining a segmental scar burden in mass and volume.

Supplementary Data 2. CMR image acquisition parameters

**Balanced steady-state free precession cine imaging:**

**Long** axis: ECG triggered with retrogating, repetition time (TR) 2.78 ms, 30 reconstructed phases, echo time (TE) 1.19ms, field of view (FOV) 340x276mm^2^, matrix 192x156, voxel size 1.8x1.8mm^2^, slice thickness (ST) 6mm, flip angle (FA) 74°, GRAPPA acceleration factor 2.

**Short axis:** ECG triggered with retrogating, TR 3.31ms, 30 reconstructed phases, TE 1.44ms, FOV 380x308.75mm^2^, matrix 192x156, voxel size 2.0x2.0mm^2^, ST 7mm, gap between slice: 0mm, FA 80°, GRAPPA acceleration factor 2.

**T2 mapping:**

Balanced steady-state free precession sequence, T2prep times 0, 25 and 55 ms, 3 recovery heartbeats, TR 2.98ms, TE 1.12ms, FA 70°, FOV 380×288mm^2^, matrix 224x170, voxel size 1.7x1.7mm^2^, ST 8mm, gap between slices 0mm, GRAPPA acceleration factor 2, motion corrected.

**T1 Mapping:**

5(3)3 MOLLI acquisition scheme, TR 3.9ms, TE 1.13ms, FA 35°, TI 180ms, FOV 360×270mm2, matrix 256x144, voxel size 1.4x1.4mm^2^, ST 8 mm, gap between slices 0mm, GRAPPA acceleration factor 2, motion corrected.

**Late gadolinium enhancement:**

PSIR reconstruction, gradient echo sequences, TI 240ms, TR 29.76ms, TE 5.17ms, FA 30°, FOV 350x263mm^2^, matrix 256x166, voxel size 1.4x1.4mm^2^, ST 7mm, gap between slices 0mm.

**Synthetic Extracellular volume:**

Prototype-sequence,

Pre-contrast: 5(3)3 MOLLI acquisition scheme, TR 3.9ms, TE 1.13ms, FA 35°, TI 180ms, FOV 360×270mm2, matrix 256x144, voxel size 1.4x1.4mm^2^, ST 6 mm, GRAPPA acceleration factor 2, motion corrected.

Post-contrast: 4(1)3(1)2 MOLLI acquisition scheme, TR 5ms, TE 1.13ms, FA 35°, TI 260ms, FOV 360×270mm2, matrix 256x144, voxel size 1.4x1.4mm^2^, ST 6 mm, GRAPPA acceleration factor 2, motion corrected.

Supplementary Data 3. Qualitative Mapping Survey

| Parameter | T1-Mapping | T2-Mapping |
| --- | --- | --- |
| Slices acquired (N=) | 1130 | 1208 |
| Slices used for analysis (N=) | 731 | 896 |
| Percentage of slices examined (%) | 64.7 | 71.9 |
| AHA-segments excluded (N=) | 85 | 14 |

Note.- data are numbers or percentages.

Supplementary Data 4. Bland-Altmann Plots for intra- and interobserver analysis


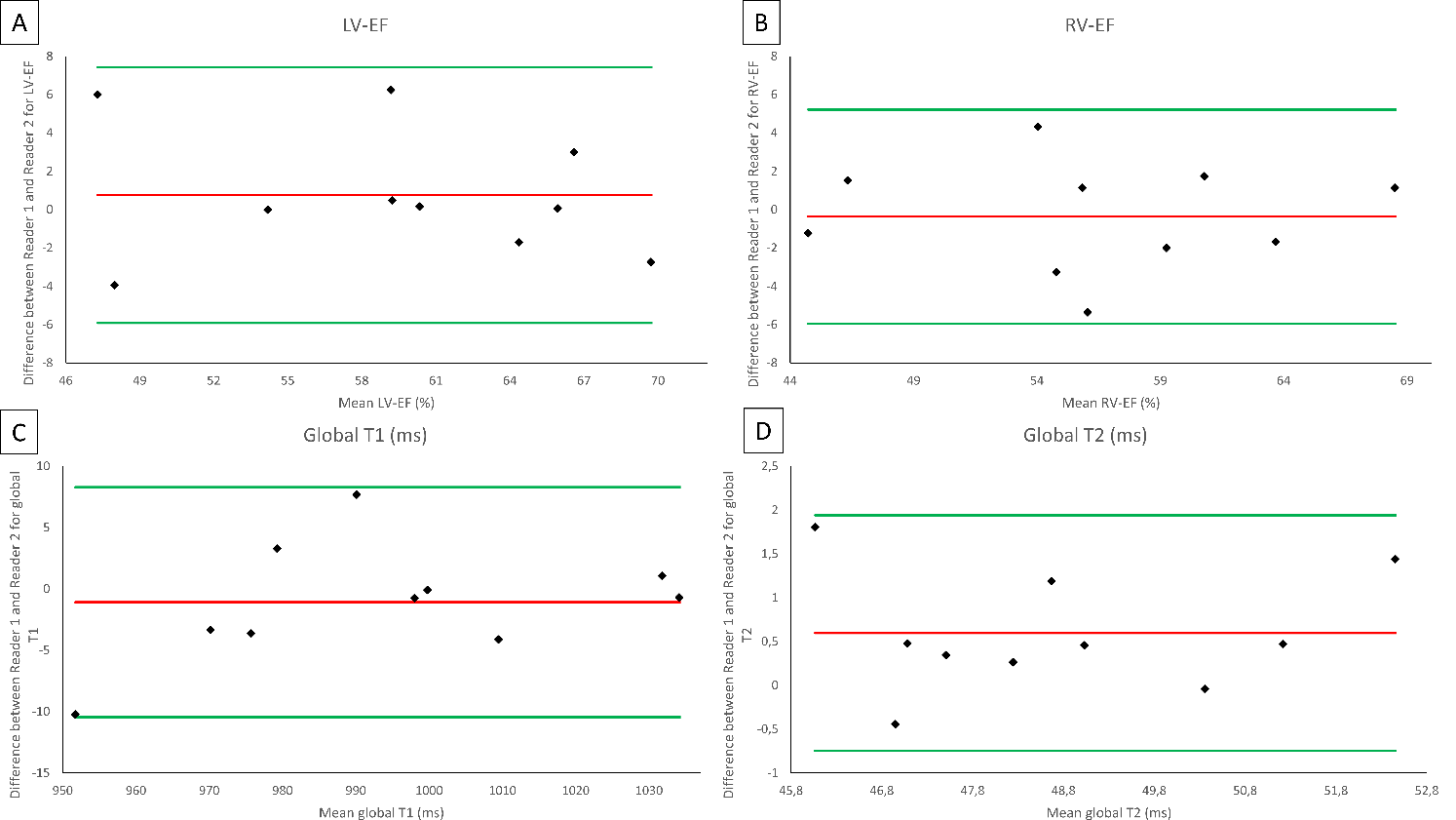


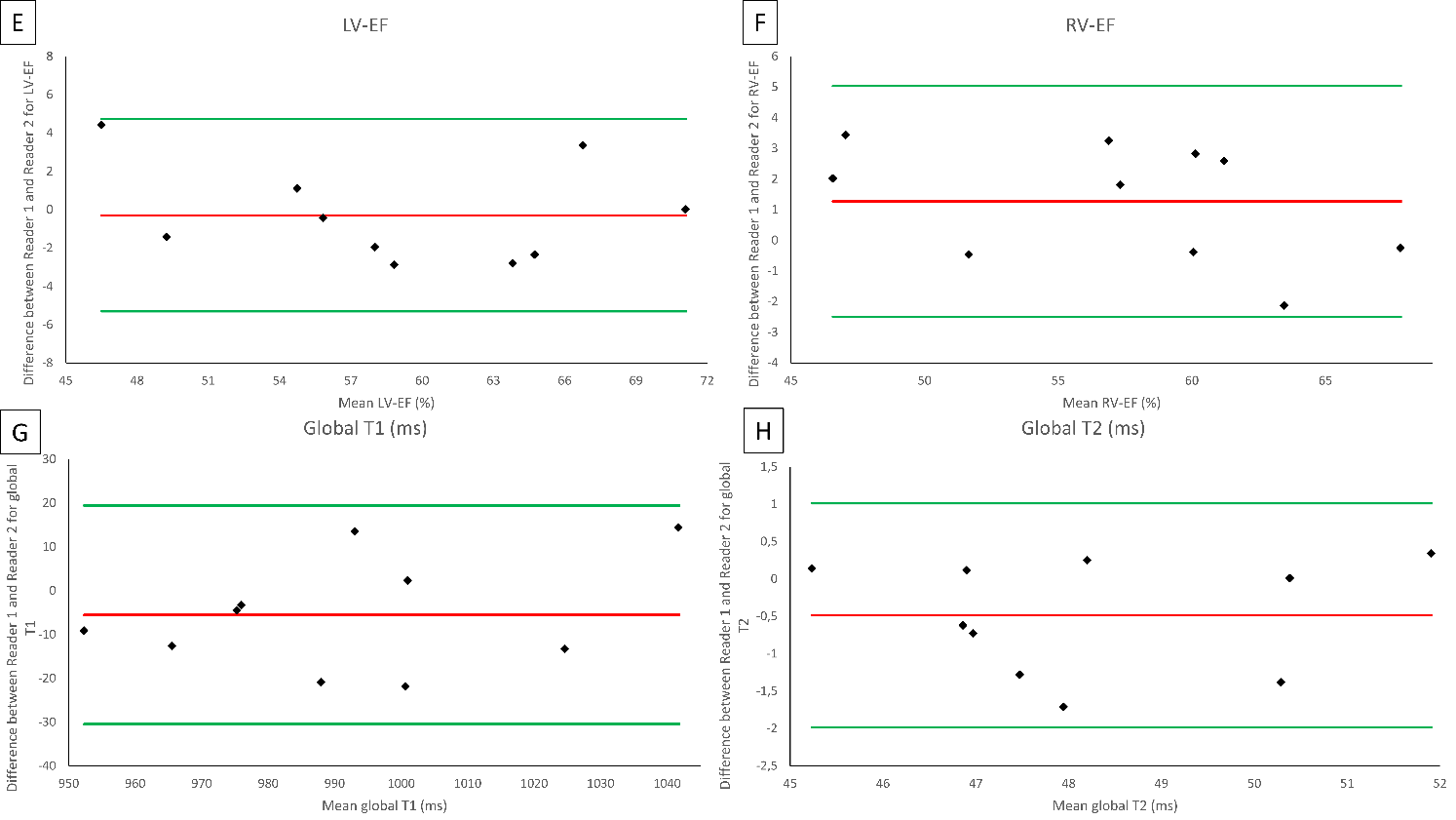


Bland-Altman analysis of intraobserver reproducibility of left ventricular ejection fraction (A), right ventricular ejection (B), global native T1 (C) and global T2 values (D). Interobserver reproducibility of left ventricular ejection fraction (D), right ventricular ejection (E), global native T1 (F) and global T2 values (G). Red line indicates mean difference; green lines indicate 95%-limits of agreement.

Supplementary Data 5. Qualitative LGE findings

Table 4: Qualitative LGE findings

| Parameter | Total (N=141) | All patients after COVID-19 infection (N=97) | Subacute COVID-19 (N=34) | Post-COVID-19 (N=63) | COVID-19 Vaccination (N=44) | p Value* | Pairings with significant differences |
| --- | --- | --- | --- | --- | --- | --- | --- |
| Total | 49 (34.6%) | 36 (37%) | 10 (29%) | 26 (41%) | 13 (30%) | .34 | n.a. |
| Ischemic pattern | 6 (4.2%) | 5 (5%) | 1 (3%) | 4 (6%) | 1 (2%) | .66 | n.a. |
| Nonischemic pattern | 43 (30.4%) | 31 (32%) | 9 (27%) | 22 (35%) | 12 (27%) | .59 | n.a. |
| Subepicardial | 22 (15.6%) | 20 (21%) | 7 (21%) | 13 (21%) | 2 (5%) | **.04** | sCov vs. CovVac; pCov vs. CovVac |
| Intramyocardial | 12 (8.5%) | 5 (5%) | 2 (6%) | 3 (5%) | 7 (16%) | .16 | n.a. |
| RV Insertionpoint | 9 (6.3%) | 6 (6%) | 0 (0%) | 6 (10%) | 3 (7%) | .17 | n.a. |

Note.- data are numbers with percentages in brackets. *p* <0.05 is considered to indicate a statistically significant difference. COVID-19= coronavirus disease of 2019; sCov= subacute COVID-19; pCov= Post-COVID-19; CovVac= COVID-19 vaccination. * *p*-values given for tests between subacute COVID-19, Post-COVID-19 and COVID-19 vaccination.
